# Evaluation of the efficiency of propofol versus isoflurane anesthesia interventions in treating elderly patients with postoperative cognitive dysfunction: A protocol for systematic review and meta-analysis

**DOI:** 10.1097/MD.0000000000023511

**Published:** 2020-12-04

**Authors:** Yan-Xi Shen, You-Ping Chen, Hong-Cheng Zang, Gang Shao

**Affiliations:** a Department of Anesthesiology, the First People's Hospital of Fuyang; b Department of Anesthesiology, the First People's Hospital of Xiaoshan, Hangzhou, Zhejiang, P. R. China.

**Keywords:** efficacy, elderly postoperative cognitive dysfunction, isoflurane, propofol

## Abstract

**Background:**

The aim of this study was to systematically evaluate the efficiency of propofol versus isoflurane anesthesia interventions in treating elderly patients with postoperative cognitive dysfunction.

**Methods:**

We performed an in-depth search in the PubMed, EMBASE, Cochrane Library, Chongqing VIP, WanFang, China National Knowledge Infrastructure, and SinoMed. Additionally, we reviewed the reference lists of included studies. Two independent authors examined the quality of the study and the quality of the extracted data. Regarding the dichotomous outcomes, we stated the results as relative risk, with 95% confidence intervals. We further expressed incessant outcomes as mean difference with a confidence level of 95%.

**Results:**

The findings of the study will be published in a peer-reviewed journal.

**Conclusion:**

Findings of this study will help in providing insight to establish if propofol is a suitable intervention to treat postoperative cognitive dysfunction in elderly patients.

**Systematic review registration number:**

INPLASY202090042

## 1 Introduction

Postoperative cognitive dysfunction is a conventional neurological complication that mainly affects elderly patients.^[1]^ It is usually clinically established as changes in patients’ mental activities, social activities, and cognitive abilities, such as memory impairment, disorientation, language impairment, and a drop in calculation abilities.^[2,3]^ This complication affects the patients’ ability to take care of themselves and causes a greater burden of care on patients’ families and society at large. Lately, however, with the increasing aging population, elderly patients have more opportunities to undergo surgery. As a result, anesthesiologists tend to pay more attention to postoperative cognitive dysfunction.

Previous reports in the literature have indicated that the prevalence of cognitive dysfunction in elderly patients within the first week following major noncardiac surgery is often as high as 25.8%.^[4]^ Meanwhile, other studies have illustrated that the occurrence of postoperative cognitive dysfunction in elderly patients is associated with anesthesia, old age, surgical methods, as well as the previous use of anticholinergic drugs before surgery. To this end, anesthesia could be associated with the incidences of postoperative cognitive dysfunction. In a study, Mason et al realized that the general anesthesia is more likely to cause postoperative cognitive dysfunction than non-general anesthesia.^[5]^ Still, the impacts of the intravenous general anesthesia, as well as, the gas general anesthesia on postoperative cognitive dysfunction remain to be controversial.^[6–8]^ For this reason, we employed a meta-analysis method to illustrate the impact of using propofol and isoflurane on elderly patients and determine the occurrence of early cognitive dysfunction after noncardiac surgery in elderly patients.

## 2 Materials and methods

The present study is listed or registered at the International Platform of Registered Systematic Review and Meta-analysis Protocols (INPLASY, https://inplasy.com/). The registration DOI of the present study is 10.37766/inplasy2020.9.0042. The protocol will be carried out under the guidelines of the Preferred Reporting Items for Systematic Review and Meta-analysis recommendations.^[9]^

### 2.1 Eligibility criteria

#### 2.1.1 Types of studies

For this present study, randomized controlled trials (RCTs), which employed propofol or isoflurane as intervention measures, were considered eligible for the research. Accordingly, the study will exclude the use of nonrandomized control studies, as well as, observational study.

#### 2.1.2 Types of participants

Furthermore, the study will consider only elderly patients (>60 years’ old) with postoperative cognitive dysfunction. The review will include participants with postoperative cognitive dysfunction, regardless of factors such as age, sex, region, or other factors. At the same time, we excluded studies that enrolled participants with heart surgery and neurosurgery.

#### 2.1.3 Types of interventions and comparisons

In the trial phase, propofol was given to the elderly patients in the treatment group and isoflurane given to elderly patients in the control group. Accordingly, there will be no limitation on the duration of trials.

#### 2.1.4 Types of outcome measures

##### 2.1.4.1 Primary outcomes

Regarding the study, the primary outcome is the occurrence of postoperative cognitive dysfunction in elderly surgical patients receiving different anesthetics.

##### 2.1.4.2 Secondary outcomes

i) Mortality at 30 days.
ii) Intraoperative hypotension as defined by the study authors.
iii) Length of stay in the postanesthesia care unit (measured in minutes).
iv) Length of hospital stay (measured as days).

### 2.2 Search methods

#### 2.2.1 Electronic searches

Furthermore, we performed an in-depth search of the following resources: PubMed, EMBASE, Cochrane Library, Chongqing VIP, WanFang, China National Knowledge Infrastructure, and SinoMed. The search was performed without language restriction. All of these databases will be correctly analyzed from the beginning to the present, with regard to their language and publication time.

#### 2.2.2 Searching other resources

To avoid the problem of missing potential studies, other sources, such as Google Scholar, will be identified to review the articles and all other primary studies to supplement the research.

#### 2.2.3 Search strategy

The search strategy was developed using a combination of “postoperative cognitive dysfunction OR POCD” AND “propofol OR isoflurane” AND “randomized clinical trial OR randomized controlled trial OR randomized OR RCT” in all fields.

### 2.3 Data collection and analysis

#### 2.3.1 Selection of studies

The above-mentioned search strategy was employed to identify titles and abstracts. From there, the selected titles and abstracts underwent screening by the 2 independent authors. From their screening, irrelevant or nonapplicable studies were discarded. However, those studies with pertinent information or data were reserved.

#### 2.3.2 Data extraction

For all the studies that were included, the 2 independent authors extracted relevant information in the data extraction phase. First, they considered necessary information valid for the present study, the first authors’ and publication dates. Second, they identified patients’ data in both the control and experimental groups, by considering the sex and age of the patients. Lastly, they used the America Society of Anesthesiologist classification, to identify measures or interventions, as well as, operations on anesthesia methods, such as anesthesia and maintenance of anesthesia, along with the right treatment or drug doses. Consulting a third author can help to resolve any disagreements between the first independent authors.

#### 2.3.3 Assessment of study quality

In particular, the quality of studies included in the present study was explored by the 2 independent authors as well to ensure compliance with the guidelines established by the Cochrane Renal Group. Obvious discrepancies were addressed by the 2 independent authors through discussion. Additionally, they considered the quality of items worth assessing by listing randomization methods, the intention of treating analysis, concealment of allocation, completeness of follow-up processes, and blinding of participants, outcome assessors, and investigators.

#### 2.3.4 Measures of treatment effect

Regarding the dichotomous outcomes, the findings of this study were stated as relative risk with 95% confidence intervals. In cases where the study considered continuous scales of measured to explore the effects of treatment with propofol, the mean difference or standardized mean difference were employed with different scales.

#### 2.3.5 Assessment of heterogeneity

Moreover, the paper analyzed heterogeneity using a *χ*^2^ test on N-1 degrees of freedom, along with an alpha value of 0.05 for the statistical significance, with the *I*^2^ test.^[10]^ In essence, *I*^2^ values of 25%, 50%, and 75% corresponded to low, medium, and high levels of heterogeneity. The paper used the fixed-effects model to estimate the effects of the amount when *P* > .1 or *I*^2^ < 50%. Also, it will use a random-effects model where the value of *P* < 0.1 or *I*^2^ > 50%.

#### 2.3.6 Assessment of reporting biases

Where >10 studies will be used, the study will use a funnel plot to identify report bias. In addition, the Egger test will be carried out to ascertain statistical investigation.^[11,12]^

#### 2.3.7 Sensitivity analysis

Overall, the article will perform a sensitivity analysis to assess the vigor of the results obtained during the study. Besides, the study will exclude studies that were incorporated in the analysis, one after another, and re-analyze, as well as, gather data to ascertain the accuracy of the results. Also, the study will evaluate the differences between the re-obtained and original effects.

### 2.4 Ethics and dissemination

For the present study, ethical approval is not warranted. Therefore, the findings of this study will only be disseminated through publishing in a peer-reviewed journal.

## 3 Discussion

Although previous studies on this topic have considered that propofol is useful for treating elderly patients with postoperative cognitive dysfunction, there are some inconsistencies in the findings of these previous studies. Additionally, most of those studies failed to employ a systematic review to evaluate the efficacy of administering propofol for treating postoperative cognitive dysfunction in elderly patients. To this end, the present study seeks to systematically examine the efficacy of propofol for the treatment of postoperative cognitive dysfunction in elderly patients. The results of this study can demonstrate evidence for clinical practice, as well as, help in health-related policy making to improve patient care for elderly patients with postoperative cognitive dysfunction.

## Author contributions

**Data curation:** Yan-Xi Shen, You-Ping Chen, and Hong-Cheng Zang.

**Formal analysis:** Yan-Xi Shen, Gang Shao.

**Investigation:** Hong-Cheng Zang.

**Methodology:** Gang Shao.

**Resources:** You-Ping Chen.

**Software:** You-Ping Chen, Hong-Cheng Zang.

**Supervision:** Yan-Xi Shen.

**Visualization:** Yan-Xi Shen.

**Writing – original draft:** yanxi shen, Gang Shao.

**Writing – review & editing:** yanxi shen, Gang Shao.

**Writing:** Yan-Xi Shen and Gang Shao.
